# ORFLine: a bioinformatic pipeline to prioritize small open reading frames identifies candidate secreted small proteins from lymphocytes

**DOI:** 10.1093/bioinformatics/btab339

**Published:** 2021-05-10

**Authors:** Fengyuan Hu, Jia Lu, Louise S Matheson, Manuel D Díaz-Muñoz, Alexander Saveliev, Jinbo Xu, Martin Turner

**Affiliations:** Laboratory of Lymphocyte Signalling and Development, The Babraham Institute, Babraham Research Campus, Cambridge CB22 3AT, UK; Laboratory of Lymphocyte Signalling and Development, The Babraham Institute, Babraham Research Campus, Cambridge CB22 3AT, UK; Laboratory of Lymphocyte Signalling and Development, The Babraham Institute, Babraham Research Campus, Cambridge CB22 3AT, UK; Laboratory of Lymphocyte Signalling and Development, The Babraham Institute, Babraham Research Campus, Cambridge CB22 3AT, UK; Laboratory of Lymphocyte Signalling and Development, The Babraham Institute, Babraham Research Campus, Cambridge CB22 3AT, UK; Laboratory of Lymphocyte Signalling and Development, The Babraham Institute, Babraham Research Campus, Cambridge CB22 3AT, UK

## Abstract

**Motivation:**

The annotation of small open reading frames (smORFs) of <100 codons (<300 nucleotides) is challenging due to the large number of such sequences in the genome.

**Results:**

In this study, we developed a computational pipeline, which we have named ORFLine, that stringently identifies smORFs and classifies them according to their position within transcripts. We identified a total of 5744 unique smORFs in datasets from mouse B and T lymphocytes and systematically characterized them using ORFLine. We further searched smORFs for the presence of a signal peptide, which predicted known secreted chemokines as well as novel micropeptides. Four novel micropeptides show evidence of secretion and are therefore candidate mediators of immunoregulatory functions.

**Availability and implementation:**

Freely available on the web at https://github.com/boboppie/ORFLine.

**Supplementary information:**

Supplementary data are available at *Bioinformatics* online.

## 1 Introduction

Open reading frames (ORFs) of <100 codons, referred to here as small ORFs (smORFs), are particularly numerous and have been challenging to annotate and to functionally characterize [reviewed in Makarewich and Olson (2017), Orr *et al.* (2020) and Yin *et al.* (2019)]. smORFs have been classified according to their location relative to the main ORF within the host transcript (Couso and Patraquim, 2017). The translation products of smORFs, termed micropeptides, have been shown to be involved in many aspects of life (D'Lima *et al.*, 2017; Huang *et al.*, 2017; Jackson *et al.*, 2018; Kondo *et al.*, 2007; Lee *et al.*, 2015; Magny *et al.*, 2013; Matsumoto *et al.*, 2017; Slavoff *et al.*, 2014). Within the immune system, the best characterized of these include host defence anti-microbial peptides, chemokines and cytokines that are known to play essential roles in normal and pathological immune reactions. A large collection of putative translatable smORFs have been identified by computational methods based on the level of DNA and protein sequence conservation across species, coding potential and context of the initiation codon. Ribosome profiling (Ribo-Seq), an approach based on deep sequencing of isolated ribosome-protected mRNA fragments, has provided extensive evidence for the translation of smORFs (Bazzini *et al.*, 2014; Ingolia *et al.*, 2009, 2014; Ji *et al.*, 2015). A variety of metrics and algorithms can use Ribo-Seq data to annotate translated regions of the genome. Amongst them ORFScore is a metric to quantify the bias of the trinucleotide periodicity pattern of ribosome-protected mRNA fragments (RPFs) towards the first reading frame in an ORF (Bazzini *et al.*, 2014). The periodicity pattern has been used by several algorithms and pipelines including ORF-RATER (Fields *et al.*, 2015), RibORF (Ji *et al.*, 2015), RiboTaper (Calviello *et al.*, 2016), RP-BP (Malone *et al.*, 2017) and RiboCode (Xiao *et al.*, 2018). Other metrics can be used in conjunction with ORFScore to improve actively translated ORF identification. For example, the ribosome release score (RRS) detects the termination of translation at the stop codon and can robustly distinguish protein-coding transcripts from ncRNAs (Guttman *et al.*, 2013). Moreover, combined Ribo-seq and RNA-seq analysis not only provides evidence for host transcripts of smORFs but also enables context-based identification of smORFs, such as during cell stress (Choudhary *et al.*, 2020; Li *et al.*, 2020; Martinez *et al.*, 2020).

Here, we describe a new analytical pipeline that we call *‘*ORFLine*’* that performs a comprehensive and systematic analysis of RNA-Seq and ribosome profiling to identify actively translated smORFs. Predicted smORFs are classified according to their host transcript type and the position of the smORF relative to other ORFs within the same transcript. We discovered 5744 actively translated smORFs during B and T cell activation and report the genetic conservation, translation efficiency (TE) and related biological processes of the predicted smORFs. We further identified smORFs containing signal peptides, some of which have the potential to be secreted.

## 2 Results and discussion

### 2.1 Overview of ORFLine

**Fig. 1.**
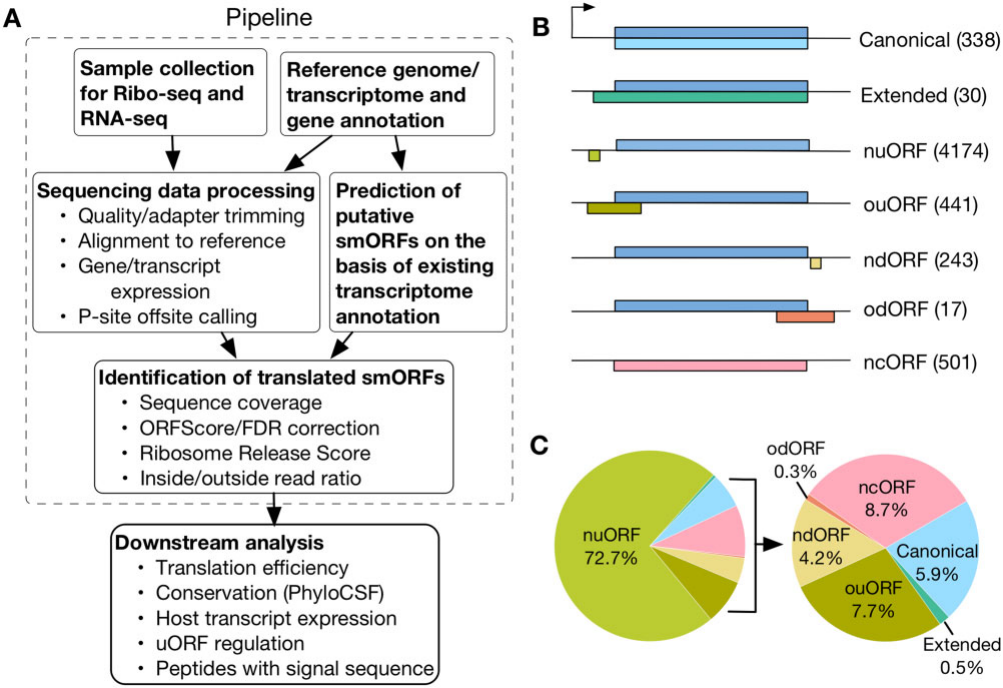
Identification of different classes of actively translated smORFs in this study. (**A**) Computational pipeline (in dashed line square) to identify translated smORFs. Sequencing data for RNA-Seq and ribosome profiling are processed and the reads mapped to the mouse reference genome GRCm38/mm10. In parallel, putative smORFs were predicted by scanning the mouse transcriptome. Several experimental metrics for each putative smORF were quantified and the smORFs exceeding a threshold for each metric were kept for downstream analysis. (**B**) Predicted smORFs were classified into seven groups according to their relative location in the host transcript. The number of smORFs in each class is shown in parentheses.nuORF, non-overlapping upstream open reading frame; ouORF, overlapping upstream ORF; ndORF, non-overlapping downstream ORF; odORF, overlapping downstream ORF; ncORF, non-coding ORF. (**C**) Pie chart showing the proportion of smORFs of different classes

ORFLine takes Ribo-Seq, RNA-Seq, reference genome, transcriptome and gene annotation as input data and produces an output list of predicted smORFs with genomic coordinates and classification (Fig. 1A). The three main pipeline components to process the raw Illumina sequences and perform smORF prediction are: (i**)** prediction of putative smORFs; (ii**)** sequencing data QC and processing; and (iii**)** identification of translated smORFs. Prediction of putative smORFs and sequencing data processing are independent components and can be executed in parallel. The identification of translated smORFs utilizes the output of the previous two components as input (Supplementary Table S1). ORFLine is applicable to data from any species.

The output of ORFLine is a list of smORFs that have passed the filters in the identification of translated smORFs. They are identified as smORFs with ribosome-protected mRNA fragments (RPF) signal. The output file (Supplementary Table S2) contains the genomic location and splicing information (including number of exons and exon lengths) of a smORF are clearly annotated and can be loaded and visualized in a genome browser. The quantitative information about a smORF is also calculated including TE, RNA expression and ribosome-protected RNA expression (FPKM value). The nucleotide sequences are retrieved and translated into amino acid sequences and presented in column 25 of Supplementary Table S2.

### 2.2 smORFs identified by ORFLine

We analysed several datasets to identify smORFs in mouse lymphocytes, including a published dataset from our lab of lipopolysaccharide (LPS)-activated B cells (Diaz-Munoz *et al.*, 2015); a new dataset of B cells activated with LPS plus interleukins IL-4 and IL-5 for 48 h; na_**ï**_ve CD4+ T cells stimulated with antibodies to CD3 and CD28, which mimics activation by antigen; and a published time-course of Th1 T cells re-stimulated with anti-CD3+anti-CD28 (Davari *et al.*, 2017) (see Supplementary Table S3). ORFLine predicted a total of 5744 unique smORFs in all samples analysed (union of 2607 smORFs predicted in B cells and 4935 smORFs predicted in T cells) (Supplementary Table S4).

ORFLine classified smORFs according to their relative position with the annotation, if any, of the host transcript (Fig. 1B). About 80% (4615) of the 5744 predicted smORFs are uORFs, whereas downstream ORFs (dORFs) is the rarest class (260; Fig. 1C). A total of 501 smORFs in putative non-coding RNAs (long non-coding RNAs and pseudogenes) were predicted, which are termed ncORF. We also detected 338 canonical smORFs, which matched exactly to annotate CDSs, and an additional 30 extended canonical smORFs, which start upstream of an annotated CDS but have the same stop codon. Of the canonical smORFs, we found direct biochemical and functional evidence for their protein products for only _**∼**_40% (135) in the UniProt protein database (Bateman, 2021) ; these smORFs include diverse entities, such as chemokines and subunits of mitochondrial complexes. The other smORF classes and the remainder of canonical smORFs (5609) have either not been functionally characterized or have not been annotated at all.

### 2.3 Comparison between ORFLine and RiboCode

We also analysed the same datasets with the recently published ORF-detection pipeline RiboCode (Xiao *et al.*, 2018), which assesses the triplet periodicity of RPFs in an ORF with modified Wilcoxon signed-rank test and has been incorporated into a recently published integrated tool (RiboToolkit) to analyse ribosome-profiling data (Liu *et al.*, 2020).

RiboCode is claimed to outperform RiboTaper, Rp-Bp and ORF-RATER (Calviello *et al.*, 2016; Fields *et al.*, 2015; Malone *et al.*, 2017). Using its default settings RiboCode predicted a total of 15 920 unique smORFs, we removed 3667 smORFs nested in longer smORFs, 48 from non-expressed transcripts and 3337 internal or frameshift smORFs. We then compared the remaining 8868 non-internal smORFs predicted by RiboCode with the 5744 predicted from ORFLine (Supplementary Fig. S2). Of these, 1957 (22.1% in RiboCode and 34.1% in ORFLine) are found as exact genomic coordinate matches by both pipelines (Table 1). We sampled the annotated smORFs, which are differentially identified by the two pipelines and noticed that smORFs predicted by RiboCode typically have low RPF coverage or are assigned a low or negative ORFScore, or low RRS, and are filtered out by ORFLine (Supplementary Fig. S3). Our criteria for metrics have shown to be robust in smORF prediction in previous studies (Bazzini *et al.*, 2014; Guttman *et al.*, 2013). ORFLine also predicted 356 smORFs encoded by low abundance transcripts (25% percentile) that are not predicted by RiboCode. Overall, RiboCode predicted more putative smORFs, but some of which appeared to share the stop codon but different start codons. RiboCode maps Ribo-Seq reads to the transcriptome and can lead to redundant positive signals from multiple transcripts. By contrast, ORFLine maps reads uniquely to the genome. We also observed that for the different putative smORFs predicted by RiboCode and ORFLine, those unique to ORFLine have higher RPF coverage and ORFScore.

**Table 1.** ORFLine and RiboCode prediction commonality/difference by class

Class	ORFLine	RiboCode	Commonality	Unique to ORFLine	Unique to RiboCode
Annotated (including canonical/ca-nonical_ exte-nded/canoni-cal_truncated)	368 (338 canonical + 30 canonical_extended)	290	183	185	107
Novel (non-coding)	501	990	69	432	921
nuORF	4174	5133	1401	2773	3732
ouORF	441	1718	185	256	1533
ndORF	243	506	121	122	385
odORF	17	231	3	14	228
Total	5744	8868	1957	3787	6911

### 2.4 smORF conservation

**Fig. 2.**
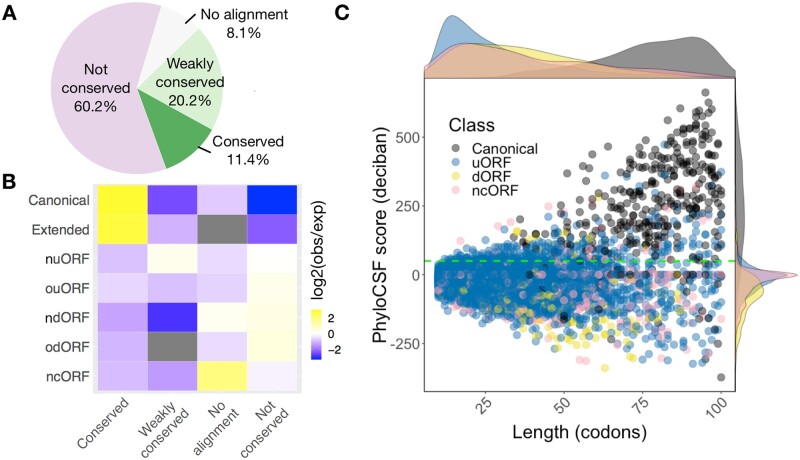
smORFs showing different conservation and length distributions according to their classes. (**A**) Most smORFs are not conserved at the peptide level. Pie chart represents the coding potential (PhyloCSF score). smORFs with PhyloCSF score ≥50 are considered conserved. smORFs are considered weakly conserved if their PhyloCSF scores are positive but smaller than the threshold 50. (**B**) Canonical and extended smORFs are enriched in conserved peptides. Enrichment heatmap depicts log 2 ratio of the number of smORF observed (obs) to the number of smORF that would be expected (exp) by chance given overall distributions of smORF classes and conservation levels. (**C**) Scatter plot shows the distributions of codon length and PhyloCSF score for each smORF type. Marginal densities of length and PhyloCSF score are also shown on the top and the right-hand side of the scatter plot. Green dashed line indicates a PhyloCSF score of 50. Here, the original classification in Figure 1B was simplified by combining the canonical and canonical extended ORFs as canonical; nuORF and ouORF as uORF; and ndORF and odORF as dORF. Canonical smORFs are on average longer and more conserved than other types

To examine the conservation of smORF-encoded micropeptides between species, we employed PhyloCSF to analyse signatures of evolutionary conservation. About 11.4% of smORFs had a PhyloCSF score >50, thus showing strong evidence of conservation (Fig. 2A), with canonical smORFs being enriched among these (Fig. 2B). A small subset (_**∼**_6.5%) of uORFs, ncORFs and dORFs showed high PhyloCSF scores, indicating conservation of smORFs CDS. There are over 60% of smORFs lacking signs of selective pressure to maintain their amino acid sequences (no cross-species sequence alignment and not conserved, Fig. 2A), in which uORFs, ncORFs and dORFs are enriched (Fig. 2B). The median length of canonical smORFs is 79 codons, however, the median length of uORF, dORF and ncORF are 24, 34 and 33 codons, respectively. By comparison with other classes, canonical smORFs are, on average, longer and more highly conserved (Fig. 2C). Having distinct transcript organization, size, conservation and peptide structure, the cellular and molecular functions of canonical smORFs, uORFs, dORFs and ncORFs are likely to differ from each other, with less conserved classes primarily independent of peptide sequences. However, we observed that the PhyloCSF score positively correlates with the length of coding sequence (data not shown). Therefore, it is likely that the conservation of shorter smORFs is underestimated.

### 2.5 Canonical smORFs

**Fig. 3.**
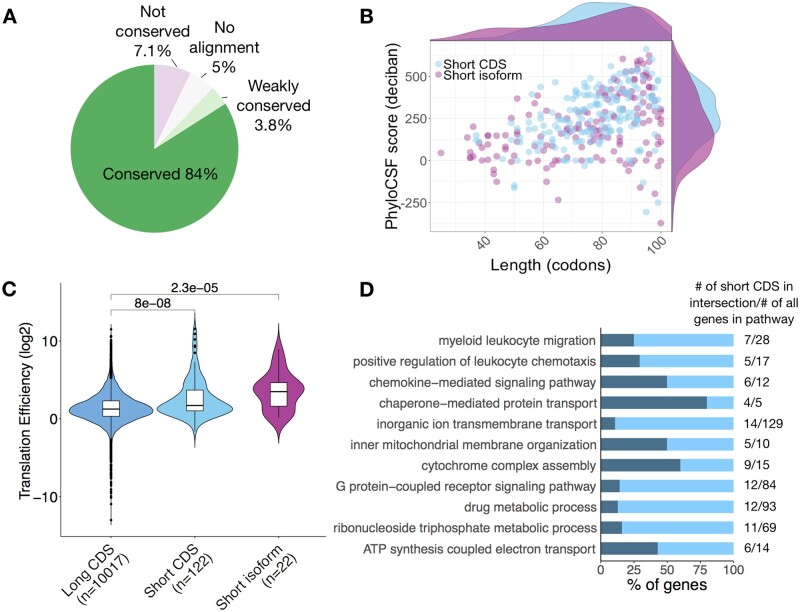
Analysis of canonical smORFs. (**A**) Pie chart shows 87.8% of canonical smORFs are conserved or weakly conserved. (**B**) Canonical smORFs were further divided to short CDSs (54.4%) and small isoforms (45.6%). A scatter plot shows the relation between ORF length and conservation score. (**C**) TE (of B cell activated with LPS and IL-4+IL5) distributions of long CDS (CDSs >100 codons), short CDSs and small isoforms. Mean and standard deviation are shown. Significance was computed using two-sided Wilcoxon test. (**D**) Biological process gene ontology terms found to be significantly enriched in the short CDS gene list

A total of 338 canonical smORFs were predicted in B and T cells. About 88% of these are conserved or weakly conserved between species (Fig. 3A). We divided canonical smORFs into *‘*short CDS*’* and *‘*short isoforms*’*, the latter are the products of alternative splicing of transcripts from genes annotated as encoding proteins longer than 100 amino acids (Couso and Patraquim, 2017). Among the predicted canonical smORFs, 54.4% are short CDSs and 45.6% are short isoforms. There are hundreds of putative short CDSs in mouse and human, these are typically located on monocistronic transcripts and their host transcripts are structurally shorter and simpler compared with canonical mRNAs (Couso and Patraquim, 2017). We have predicted 184 short CDSs and they have a median size of 79 codons. We find short isoforms have a median size of 80 codons and resemble short CDSs in size and conservation (Fig. 3B). As short isoforms share conserved protein sequences with their longer canonical protein isoforms, they may have functions that are directly related to their longer protein isoforms (Couso and Patraquim, 2017).

To increase confidence that predicted canonical smORFs were indeed translated, we calculated the TE of the short CDSs and short isoforms. When compared to long CDSs of expressed protein-coding transcripts, we found their median TE to be greater (Fig. 3C). We also conducted GO term enrichment analysis comparing the 184 short CDS and the 154 short isoforms against all smORF-encoding genes of B and T cells. The top hits of short CDS are related to chemokine activity and mitochondrial biology (Fig. 3D and Supplementary Table S5). We observed enrichment of gene products involved in mitochondrial complexes: e.g. Romo1 is located in the mitochondrial membrane and is responsible for increasing the level of reactive oxygen species in cells (Na *et al.*, 2018). Romo1 also has anti-microbial activity against a variety of bacteria by penetrating the bacterial membrane (Sha *et al.*, 2012). The chemokines Ccl1, Ccl22, Ccl3, Ccl4, Ccl5, Cxcl10 and Cxcl11 are predicted indicating the ability of the pipeline to identify *bona fide* micropeptides with immunoregulatory properties.

### 2.6 uORFs

**Fig. 4.**
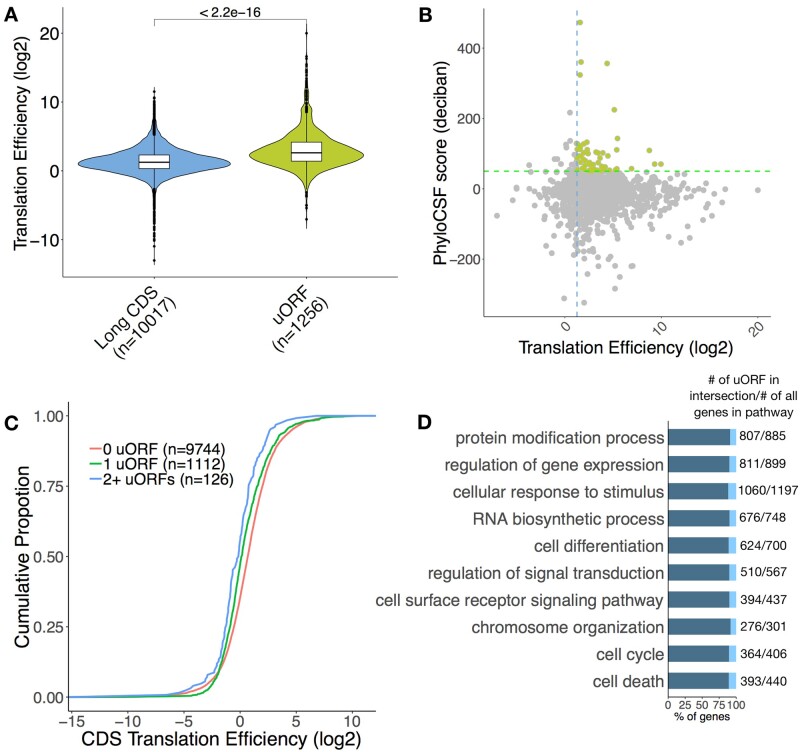
uORFs regulate the translation of their downstream CDS. (**A**) TE distributions of long CDS and uORF. Significance was computed using a two-sided Mann–Whitney test. (**B**) Scatter plot of uORF TE and PhyloCSF score. Green broken line represents a PhyloCSF score of 50 used as a threshold for conservation, blue broken line represents the median TE of long CDS. uORFs that are conserved and have a high TE are highlighted. (**C**) Cumulative distribution of TE in expressed uORF-containing transcripts versus transcripts lacking uORFs as control. Significance was computed using two-sample Kolmogorov–Smirnov test for each uORF set compared to the control (1 uORF *P*=1.321e-14, 2+ uORFs *P*=1.828e-6). (**D**) Biological process gene ontology terms found to be significantly enriched in the uORF-containing gene list

Approximately 50% of annotated animal mRNAs contain uORFs (Andrews and Rothnagel, 2014; Couso and Patraquim, 2017) and translation of uORFs has been widely reported in different organisms (Calvo *et al.*, 2009; Johnstone *et al.*, 2016; Wang and Rothnagel, 2004). We observed that the median TE of uORFs is greater than that of long CDS (Fig. 4A). About 4% of the uORFs have a high PhyloCSF score and TE above the median TE of long CDS and potentially encode conserved functional micropeptides (Fig. 4B, for B cells activated with LPS and IL-4+IL-5). However, the amino acid sequences of the majority of uORFs are not conserved, suggesting that any potential function is largely independent of the encoded peptide. The proportion of expressed uORF-containing transcripts in B cells and T cells is between 6.2% and 12.4%, except resting B cells (2.7%). Several studies have shown a repressive effect of uORFs on the translation of the main CDS (Chew *et al.*, 2016; Johnstone *et al.*, 2016; Zhang *et al.*, 2019). We compared the TE of the CDS in all uORF-containing transcripts versus those lacking uORFs. As expected, the presence of uORFs and overlapping uORFs was associated with a translation repression (Fig. 4C). We performed GO enrichment analysis for all uORF-containing genes to discover their associated biological processes (2881 target genes) and these genes are mostly enriched in processes linked to protein modification, regulation of gene expression and cellular response to stimulus (Fig. 4D and Supplementary Table S5). These enrichments are consistent with regulatory uORFs mediating the rapid changes in gene expression in response to stress and environmental stimuli.

### 2.7 smORFs in non-coding RNAs

**Fig. 5.**
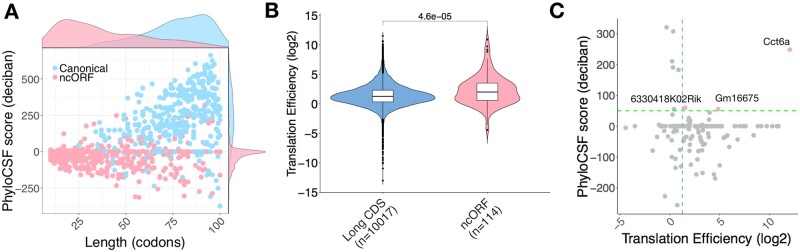
Translated smORFs predicted in non-coding RNAs. (**A**) Canonical smORFs and ncORFs showing very different distributions in length and PhyloCSF score. (**B**) TE distributions of long CDS and ncORF. Significance was computed using a two-sided Mann–Whitney test. (**C**) TE and PhyloCSF score are shown for ncORFs (LPS-activated B cells). Scatter plot of ncORF TE and PhyloCSF score. Green broken line represents a PhyloCSF score of 50 used as a threshold for conservation, blue broken line represents the median TE of long CDSs. ncORFs that are conserved and have high TE are highlighted. Three genes (Cct6, 6330418K02Rik, Gm16675) potentially encode micropeptides

Non-coding ORFs (ncORFs) are smORFs that are found in annotated long non-coding RNAs (lncRNAs) and pseudogenes. They are typically short with a median length of 33 codons. By definition, non-coding RNAs are not translated into protein. However, annotated lncRNAs have been predicted from their sequences to contain six smORFs on average (Couso and Patraquim, 2017). We have predicted 501 translated ncORFs and 14.4% of these are considered conserved or weakly conserved. We noticed very different distributions of size and PhyloCSF score between ncORFs and canonical smORFs (Fig. 5A). The distribution of TE for ncORFs is also different from that for long CDS, the median TE of ncORFs is greater than long CDS (Fig. 5B). Three ncORFs identified in LPS-activated B cells (Cct6a, Gm16675 and 6330418K02Rik) were found to have a high PhyloCSF score and TE, so we infer them to encode functional micropeptides (Fig. 5C). We searched the micropeptides they encode in NCBI BLASTp database (Altschul *et al.*, 1990), but did not find any match for Gm16675 and a partial match for 6330418K02Rik to three uncharacterized proteins with 35.59–78.18% identity in *Habropoda laboriosa* and *Gulo gulo*. The examples likely reflect that these genes are misclassified as non-coding, although it is possible that they could also have functions as a non-coding RNA in addition to their peptide coding capacity.

### 2.8 dORFs

A total of 243 ndORFs and 17 odORFs were predicted with a median length of 34 AA. Only 20 (_**∼**_7.7%) are conserved or weakly conserved (Supplementary Table S6). The TE of dORFs is lower than the long CDSs in general (data not shown). The low TE indicates a very low level of translational reinitiation after the stop codon of the upstream CDS (Gunisova *et al.*, 2018). In B cells activated with LPS and IL-4 + IL-5, we noticed that dORF-containing transcripts show no significant difference in TE compared to those without (Supplementary Fig. S4).

### 2.9 Signal sequence-containing micropeptides

**Fig. 6.**
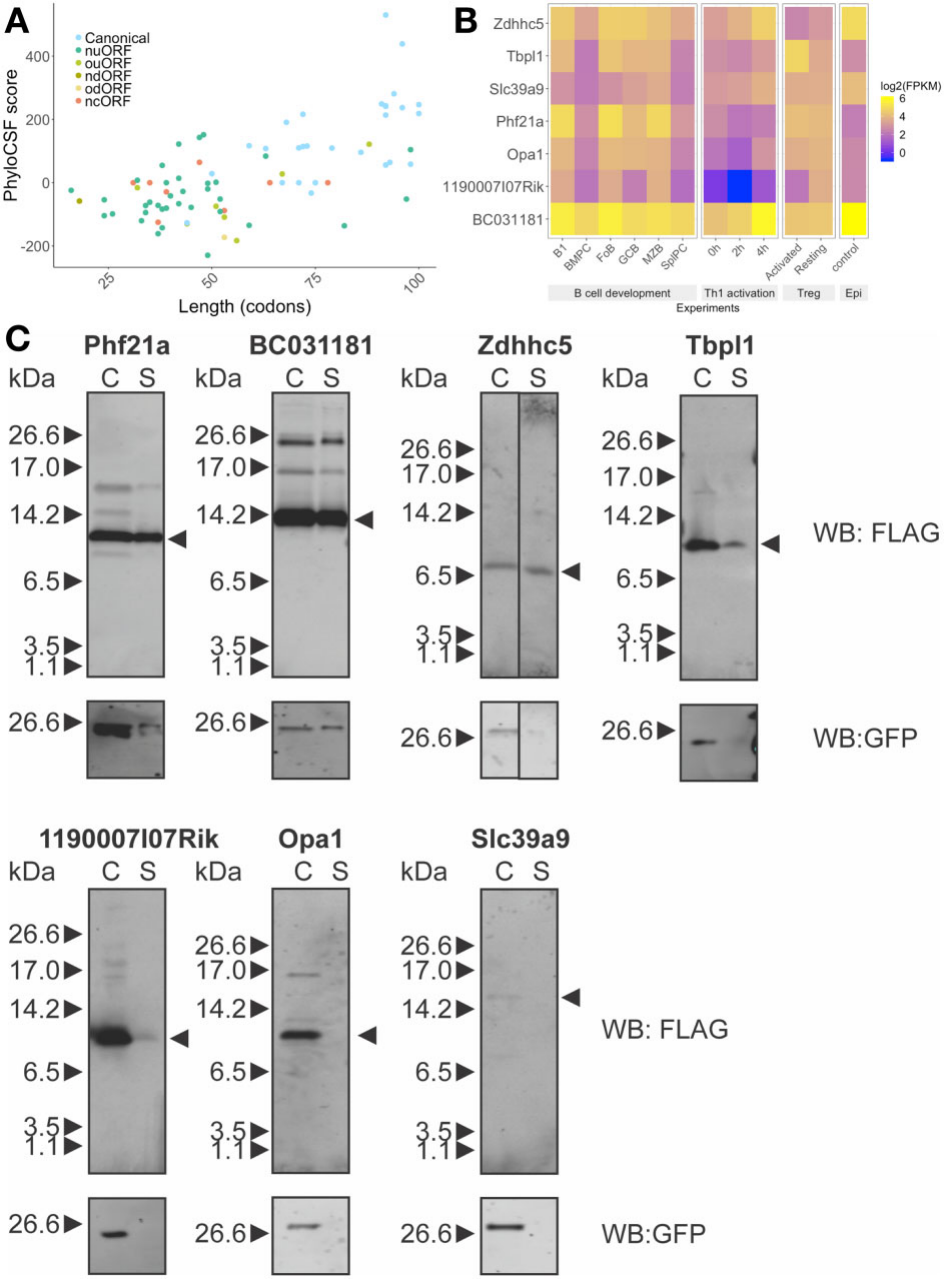
Predicted signal sequence-containing micropeptides and their host transcripts expression under different conditions. (**A**) Scatter plots show the distributions of length (codon) and PhyloCSF score for each predicted signal peptide containing micropeptides. (**B**) Heatmap analysis of host transcript expression during B cell terminal differentiation, Th1 cell activation, resting/activated regulatory T cells and epidermal cells (Epi). Selected micropeptides are shown in the heatmap, they are conserved in humans and there is limited or no information regarding their function. They are ordered by length. (**C**) Expression and secretion of micropeptides. Plasmids encoding predicted micropeptides were transfected into 293 T cells and micropeptides in total cell lysate (C) and total secreted fraction (S) were detected by anti-FLAG antibody. GFP expression indicates transfection efficiency

An N-terminal signal peptide sequence of 16–30 amino acids is characteristic of proteins destined to be secreted, resident within cellular membranes or within compartments of the secretory pathway. Using SignalP server (Petersen *et al.*, 2011), we predicted 80 candidates including known chemokines (CCL-1, -2, -4, -5 and -22) and the cell surface protein CD52, as well as the recently identified lncRNA encoded Aw112010 (Jackson *et al.*, 2018) and 1810058I24Rik micropeptides (Bhatta *et al.*, 2020). Amongst these, 28 are canonical micropeptides and they typically have high levels of conservation. Of the remaining 52 non-canonical micropeptides, 12 show conservation (Fig. 6A).

We selected seven putative smORFs for further characterization (Fig. 6B). First of all, all of the selected uORFs do not overlap with the main CDS. Except for Zdhhc5, all are in different reading frames from the main CDS. To examine the expression of signal sequence-containing micropeptide host transcripts, we compared mouse RNA-Seq datasets for lymphocytes spanning B cell terminal differentiation (Shi *et al.*, 2015), Th1 cell activation (Davari *et al.*, 2017) and regulatory T cells (Luo *et al.*, 2016) as well as epidermal cells (Sendoel *et al.*, 2017). These data revealed dynamic expression patterns for several of the host transcripts. For example, BC031181 was down-regulated during B cell differentiation but up-regulated during Th1 cell activation, it was also highly expressed in epidermal cells (Fig. 6B). To determine if the selected putative micropeptides are likely functional, we compared the conservation of amino acid sequence between different mammalian species (Supplementary Fig. S5). All of the micropeptides including those encoded in uORFs show evidence of conservation. This indicates positive selection pressure of the coding sequence of these micropeptides.

### 2.10 *In vitro* characterization of predicted micropeptides with signal sequence

We investigated if the micropeptides with signal sequences are secreted. To this end, we selected and cloned seven putative smORFs with predicted signal sequences into a dicistronic mammalian expression vector, which allowed synthesis of the micropeptide with a FLAG epitope tag at its C-terminus and of GFP driven by an IRES from the same transcript. HEK293T cells transfected with micropeptide-encoding plasmids displayed anti-FLAG signals in both total cell lysates (C) and supernatant (S) fractions (Fig. 6C). GFP was detected in all total cell lysates, but the smORF encoded in the Slc39a9 transcript showed no evidence of expression. The smORFs encoded by Phf21a (uORF) and BC031181 (canonical) showed the most abundant expression and secretion. The smORFs encoded by Zdhhc5 and Tbpl1 expressed less strongly but showed evidence of secretion. By contrast, the smORFs encoded by Opa1 and 1190007I07Rik were weakly or not at all secreted (Fig. 6C). A recent report demonstrates that the human ortholog of 1190007I07Rik, named *C12orf73*, encodes a functional micropeptide named BRAWNIN found at the inner mitochondrial membrane and required for respiratory complex III assembly (Zhang *et al.*, 2020). These results demonstrate that putative smORFs can be expressed and secreted, but additional investigations are required to validate their subcellular localization and to demonstrate their biological functions.

Further investment into the generation of antibodies and model organisms will be required to assign function to micropeptides with signal sequences. It will be exciting to validate the existence of receptors and to shed light onto the biology of these micropeptides.

## Supplementary Material

btab339_supplementary_data
