# Correction to: ORFLine: a bioinformatic pipeline to prioritize small open reading frames identifies candidate secreted small proteins from lymphocytes

**DOI:** 10.1093/bioinformatics/btac162

**Published:** 2022-03-29

**Authors:** Fengyuan Hu, Jia Lu, Louise S Matheson, Manuel D Díaz-Muñoz, Alexander Saveliev, Martin Turner

*Bioinformatics*, 2021, 37(19), 3152–3159. https://doi.org/10.1093/bioinformatics/btab339

In the originally published version of this manuscript, the Associate Editor’s name, Jinbo Xu, was listed as an author in error. The publisher apologizes for the error. This error has been corrected online.

