# Supplementary material for: ORFLine: a bioinformatic pipeline to prioritize small open reading frames identifies candidate secreted small proteins from lymphocytes
Source: Bioinformatics. 2021 May 10;37(19):3152–9. doi: 10.1093/bioinformatics/btab339 (PMC8504629; doi:10.1093/bioinformatics/btab339)
Supplement: btab339_supplementary_data [file btab339_supplementary_data.zip › Supplementary Data.doc]

**Supplemental tables and figures:**

**MATERIALS AND METHODS**

**Tissue culture**

B lymphocytes from the spleen or lymph nodes (LNs) of C57BL/6 mice were isolated using the B Cell Isolation Kit (Miltenyi Biotec). For activation, B cells were cultured for 48 hours in RPMI 1640 Medium (Dutch Modification) supplemented with 10% FCS, 100 IU/ml penicillin, 100 μg/ml streptomycin, 2 mM L-GlutaMAX (Gibco), 1 mM Sodium Pyruvate and 50 μM β-mercaptoethanol in the presence of 10 μg/ml of LPS (Sigma, E. Coli 0127: B8), 10ng/ml of IL4 and 10ng/ml of IL5. Naïve CD4+ T lymphocytes from spleen and LNs were isolated with CD4+CD62L+ T Cell Isolation Kit (Miltenyi Biotec) and stimulated in the same medium as for B cells using plate bound anti-CD3 (2C11) and 1 μg/ml of anti-CD28 (37.51) for 24 hours. HEK293T cells were maintained in DMEM (Gibco) with 10% FBS (Gibco) and 1× GlutaMAX-I (Gibco).

**cDNA Library preparation** RNA-Seq libraries were generated using the TruSeq Stranded mRNA Sample Prep Kit (Illumina Inc). Ribo-Seq libraries were prepared using the ARTseq^™^ Ribosome Profiling Kit (Epicentre, Illumina) from cells treated with cycloheximide (CHX, 100 μg/ml) prior to cell lysis. cDNA libraries were sequenced using Illumina HiSeq1000, Illumina HiSeq2000 or Illumina HiSeq2500 system in a 100-bp single-end (RNA-Seq) or 50-bp single-end (Ribo-Seq) mode. Summary metrics of libraries used are described in **Table S3**.

**Reference genome, transcriptome and annotation**

GENCODE (Harrow, et al., 2012) reference genome sequences (mouse GRCm38/mm10) are downloaded from the GENCODE website (**Table S8**). Transcriptome sequences (cDNA sequences) and gene annotation downloaded from the same GENCODE source are used to search for putative ORFs. tRNA sequences are downloaded from UCSC Table Browser (Karolchik, et al., 2004). rRNA sequences are downloaded from GENCODE (version M20, we have also tested M13 and M15) as well as published studies (Bazzini, et al., 2014; Fields, et al., 2015). The transcriptome is defined as the collection of all transcripts on the reference chromosomes. GENCODE Transcript biotypes are defined here - <https://www.gencodegenes.org/pages/biotypes.html>. In our pipeline, we remove the following biotypes:

- IG_* and TR_* (Immunoglobulin variable chain and T-cell receptor genes)
- miRNA
- misc_RNA
- Mt_rRNA and Mt_tRNA
- rRNA and ribozyme
- scaRNA, scRNA, snoRNA, snRNA and sRNA
- nonsense_mediated_decay
- Non_stop_decay

**Sequencing data processing**

Raw Illumina sequencing data in FASTQ format are trimmed of adapter sequences and the resultant reads are aligned against specific sequences assembled from a collection of rRNA, tRNA, Mt_rRNA and Mt_tRNA snRNA, snoRNA, misc_RNA and miRNA sequences using Bowtie v1.2.2 (Langmead, et al., 2009) to remove these sequences. The remaining reads are aligned to the reference genome (GRCm38). Adaptor trimming and quality trimming (including poor quality “N” base at the 5’ end of some of the reads) were performed with Trim Galore v0.4.5 (https://www.bioinformatics.babraham.ac.uk/projects/trim_galore), quality checked with FastQC v 0.11.8 (<http://www.bioinformatics.bbsrc.ac.uk/projects/fastqc>). Authentic RPFs will be ~28-30nt in length, therefore, we have kept trimmed reads that have a length between 25 and 35 nt, as they account for ~75% of the total reads on average (refer to information in **Table S3**).

**Alignment to reference genome:** The reads were mapped to the GRCm38/mm10 reference genome using the STAR aligner v2.5.2a (Dobin, et al., 2013). The aligner reports only uniquely mapped reads (mapping quality MAPQ = 255). The following shows an example command, and parameters are in bold:

STAR **--runThreadN** $THREAD \

**--genomeDir** $REFGENOMESTAR \

**--readFilesIn**$OUTPATH/bowtie-contanminant-removal/${NAME}_trimmed_unfiltered.fq.gz

**--readFilesCommand** zcat \

**--outReadsUnmapped** Fastx \

**--outFileNamePrefix** $OUTPATH/star-genome/$NAME/ \

**--alignIntronMin** $ALIGNINTRON_MIN \

**--alignIntronMax** $ALIGNINTRON_MAX \

**--alignEndsType** EndToEnd \

**--outFilterMismatchNmax** $MISMATCH_MAX \

**--outFilterMismatchNoverLmax** $MISMATCH_NOVERL_MAX \

**--outFilterType** $FILTER_TYPE \

**--outFilterIntronMotifs** RemoveNoncanonicalUnannotated \

**--outSAMattributes** $SAM_ATTR \

**--outSAMtype** BAM SortedByCoordinate \

**--outBAMsortingThreadN** $THREAD

**Transcript expression quantification:** In each experiment, sequence alignments (in BAM format) of all biological replicates were combined for RNA-Seq and Ribo-Seq respectively. Transcript expression was quantified using StringTie v1.3.6 (Pertea, et al., 2015) in FPKM (Fragments Per Kilobase per Million). From a given dataset, a minimal expression level was set to FPKM > 0.5 (Hart, et al., 2013) to exclude non-expressed transcripts.

**P-site offset determination:** A majority of RPFs has a length between 28-31 nucleotides (nt). The 5’ P-site offset is the distance from the 5’ end of the read to the ribosomal P-site (Ingolia, et al., 2009). To determine P-site offset, we separated footprints into groups based on their lengths, P-site offset was estimated for each read length using plastid python library v0.4.8 (Dunn and Weissman, 2016). We observed P-site offsets are 12 nt long for RPF in 28-31 nt in our experiments.

**Cloning and expression of candidate secreted micropeptides:** The coding sequences of predicted smORFs were codon optimised for mammalian expression (GeneArt from ThermoFisher) and cloned into a mammalian expression vector. A C-terminal FLAG epitope tag was placed downstream of the micropeptide cDNA sequences, followed by an EMCV-IRES and GFP cDNA. The di-cistronic mRNA is controlled by a CAG promoter. For transfection, Trans-IT (Mirus Bio) was used to deliver 1 μg plasmid to HEK293T cells per well of 6-well plates according to the manufacturer’s instruction. 21-24 hours post transfection, cells were separated from total secreted proteins by centrifugation at 300 x g for 5 minutes at 4 ℃ and were lysed using RIPA buffer. Total cell lysates and total supernatant were resolved with 16% Tris-Tricine/SDS-PAGE (Haider, et al., 2019) and were blotted with anti-FLAG (clone M2, Sigma) and anti-GFP (clone D5.1, Cell Signaling Technology) antibodies. Images were acquired and analysed using the Odyssey CLx (LI-COR).

**Overview of strategy to identify candidate smORFs**

Using the nucleotide sequences of all transcripts downloaded from GENCODE (release M13) (Frankish, et al., 2015) as a reference, ORFLine searches for putative ORFs beginning with “NUG” and ending with "UAG", "UAA", "UGA" in each of the three reading frames. It then removes ORFs that are not n*3 (n > 1) nucleotides long and designates those that are between 10 to 100 codons in length as putative smORFs. The ORF coordinates are initially transcript coordinates and are converted to genomic coordinates given exon location information in the gene annotation (in GTF/GFF format), the output of this step are genomic coordinates and strands for putative smORFs in BED format. Each ORF will be assigned two different identifiers, one is called RegionId, the second is called ORFId. RegionId is created based on genomic coordinates, ORFId is created based on the transcript coordinates. An ORF has a unique genomic location, thus RegionId is unique, but it may arise from multiple overlapping transcripts, so it may have multiple ORFIds (**Figure S1**). This step is carried out only once and needs to be updated when transcriptome annotation is changed.

**Identification of translated smORFs**

ORFLine takes the gene annotation, putative smORFs, Ribo-Seq and RNA-Seq alignment as input to predict actively translated smORFs. ORFLine combines alignment files of all biological replicates (pooled analysis) to increase the signal intensity in case the smORFs are lowly expressed. This component consists of several metrics and filters, putative smORFs that have exceeded a confidence threshold for each metric (as indicated in **Table S1**) were kept.

**RPF coverage:** To filter ORFs which are insufficiently covered by reads, we calculated the proportion of codons being covered by RPFs. We consider a codon covered if there is a mapped RPF with the P-site aligned to nucleotide 1 of that codon. An ORF is discarded if the ratio of covered codons to the total number of codons in the ORF < 0.1 (Bazzini, et al., 2014).

**ORFScore:** ORFScore was proposed by Bazzini and colleagues (Bazzini, et al., 2014) and we re-implemented the ORFScore algorithm in R. The ORFScore was then calculated as:

Where_n_ is the number of reads in reading frame n, is the total number of reads across all three frames divided by 3. RPFs were counted at each position within an ORF, excluding the first and last coding codons. To filter out putative artifactual peaks, the most abundant read position was masked if reads aligning to that position comprised more than 70% of the total reads in the ORF. The ORFScore is a log-scaled chi-squared goodness of fit test statistic, the p-values associated with the test were adjusted using Benjamini-Hochberg FDR-controlling method and smORFs with ORFScore > 0 and adjusted p-value < 0.01 were retained.

**Ribosome Release Score (RRS):** Firstly, the 3’UTRs of smORFs is defined. For canonical smORFs, we used annotated 3’UTRs. For other classes of smORFs, their 3’UTRs were defined as the region between the stop codon and the next possible start codon in any frame. The RRS score is defined as the ratio of the two normalized ratios and calculated with the following equation: RRS = (FPKM_RF ORF/FPKM_RF 3’-UTR)/ (FPKM_RNA ORF/FPKM_RNA 3’-UTR). Based on the original study, smORF with RRS > 5 is considered to be translated (Guttman, et al., 2013).

**Inside/outside read ratio**: The ribosome footprints typically show precise positioning between the start and the stop codon of translated ORFs. Low density of footprints before start codons and after stop codons and high inside/outside ratio is expected. By considering the read distribution of the nearest 3 upstream codons outside and the first 3 codons inside an ORF, we used a feature called inside/outside read ratio (total RPF of inside 3 codons/total RPF of outside 3 codons) to assess whether genuine translation takes place. ORFs will be discarded if the ratio ≤ 1 (more reads mapping outside than inside).

**Analysis of predicted smORFs**

**Translation efficiency (TE)**: A measure of the rate of translation for a given feature (e.g. the CDS of a mRNA or a smORF), obtained in ribosome profiling experiments. It was calculated as the base 2 logarithmic ratio of RPF expression (FPKM) over mRNA expression (FPKM).

**Conservation of the amino acid sequences**: To examine the conservation of smORF-encoded micropeptide sequences between species, we performed PhyloCSF (Lin, et al., 2011), a likelihood-based method to analyse signatures of evolutionary conservation in multiple species sequence alignments (https://github.com/mlin/PhyloCSF/wiki#available-phylogenies). For each smORF, we selected alignments of mouse, human, chimpanzee, gorilla, cow, dog and zebrafish from a publicly available whole genome multiple alignment using Galaxy “stitch gene blocks” tool (Blankenberg, et al., 2011). smORFs were considered conserved if their PhyloCSF score was > 50 (Guttman, et al., 2013), and weakly conserved if they had a PhyloCSF score > 0. PhyloCSF score = 0 indicates that there is no DNA sequence alignment cross species and PhyloCSF score < 0 is considered not conserved.

**Gene ontology (GO) enrichment analysis**: We used the g:Profiler server (Raudvere, et al., 2019) to perform GO analysis in two unranked lists of genes mode. The background list comprised all smORF host transcripts from B and T cells. The target list contains the host transcripts of specific classes of smORF. For the significance threshold, we chose the default option g:SCS threshold and the default value 0.05.

**Secreted micropeptide prediction**: We used the SignalP 4.0 server (Petersen, et al., 2011) to predict signal peptides present at the [N-terminus](https://en.wikipedia.org/wiki/N-terminal_end) of the micropeptide amino acid sequences. We used default parameters. For selected candidates, we ran prediction of transmembrane helices using the TMHMM 2.0 Server (Krogh, et al., 2001) (default parameters) to rule out transmembrane peptides.

**References**

Bazzini, A.A.*, et al.* Identification of small ORFs in vertebrates using ribosome footprinting and evolutionary conservation. *The EMBO journal* 2014;33(9):981-993.

Blankenberg, D.*, et al.* Making whole genome multiple alignments usable for biologists. *Bioinformatics* 2011;27(17):2426-2428.

Dobin, A.*, et al.* STAR: ultrafast universal RNA-seq aligner. *Bioinformatics* 2013;29(1):15-21.

Dunn, J.G. and Weissman, J.S. Plastid: nucleotide-resolution analysis of next-generation sequencing and genomics data. *BMC Genomics* 2016;17(1):958.

Fields, A.P.*, et al.* A Regression-Based Analysis of Ribosome-Profiling Data Reveals a Conserved Complexity to Mammalian Translation. *Mol Cell* 2015;60(5):816-827.

Frankish, A.*, et al.* Comparison of GENCODE and RefSeq gene annotation and the impact of reference geneset on variant effect prediction. *BMC Genomics* 2015;16 Suppl 8:S2.

Guttman, M.*, et al.* Ribosome profiling provides evidence that large noncoding RNAs do not encode proteins. *Cell* 2013;154(1):240-251.

Haider, S.R., Reid, H.J. and Sharp, B.L. Tricine-SDS-PAGE. *Methods in molecular biology* 2019;1855:151-160.

Harrow, J.*, et al.* GENCODE: the reference human genome annotation for The ENCODE Project. *Genome Res* 2012;22(9):1760-1774.

Hart, T.*, et al.* Finding the active genes in deep RNA-seq gene expression studies. *BMC Genomics* 2013;14:778.

Ingolia, N.T.*, et al.* Genome-wide analysis in vivo of translation with nucleotide resolution using ribosome profiling. *Science* 2009;324(5924):218-223.

Karolchik, D.*, et al.* The UCSC Table Browser data retrieval tool. *Nucleic Acids Res* 2004;32(Database issue):D493-496.

Krogh, A.*, et al.* Predicting transmembrane protein topology with a hidden Markov model: application to complete genomes. *J Mol Biol* 2001;305(3):567-580.

Langmead, B.*, et al.* Ultrafast and memory-efficient alignment of short DNA sequences to the human genome. *Genome Biol* 2009;10(3):R25.

Lin, M.F., Jungreis, I. and Kellis, M. PhyloCSF: a comparative genomics method to distinguish protein coding and non-coding regions. *Bioinformatics* 2011;27(13):i275-282.

Pertea, M.*, et al.* StringTie enables improved reconstruction of a transcriptome from RNA-seq reads. *Nature biotechnology* 2015;33(3):290-295.

Petersen, T.N.*, et al.* SignalP 4.0: discriminating signal peptides from transmembrane regions. *Nat Methods* 2011;8(10):785-786.

Raudvere, U.*, et al.* g:Profiler: a web server for functional enrichment analysis and conversions of gene lists (2019 update). *Nucleic Acids Res* 2019;47(W1):W191-W198.

**Table S1.** Summary of steps in the ORFLine.

**Table S2.** Pipeline final output format.

**Table S3.** Sequencing metrics.

**Table S4.** Predicted smORFs. The columns are Region ID, Chromosome, Start, Stop, Strand, Class, Transcript ID (Ensembl), Gene symbol, Gene description, PhyloCSF score, AA length, Peptide sequence, Cell types.

**Table S5.** GO enrichment.

**Table S6.** dORF conservation.

**Table S7.** Public datasets used in this study.

**Table S8.** Reference annotation. List of public sequences and annotation files used in the pipeline. Reference genome and transcriptome sequences, gene annotation (mouse and human) are from GENCODE. Ribosomal RNA (rRNA) and Transfer RNA (tRNA) sequences are from UCSC Table Browser. rRNA sequences are from Ensembl.

**Figure S1.** RegionId and ORFId explained. RegionId is genomic-based, it indicates the unique location of a smORF on the genome. ORFId is transcript-based, it contains information regarding the smORF’s relative position on its host transcript.

**Figure S2**. Number of smORFs used for a comparison between RiboCode and ORFLine. Initially, 15920 smORFs were predicted by RiboCode, 3367 were removed as they were nested in longer smORFs in the same frame. 48 were removed as they were from non-expressed host transcripts, and 3337 were removed as they were internal smORFs. The remaining 8868 were used to compare with ORFLine prediction.

**Figure S3.** ORFLine predicts putative smORFs with more robust metrics. The following metrics of smORFs differentially predicted by ORFLine and RiboCode were compared: A) Read count B) RPF coverage C) ORFScore and D) RRS. Green dotted lines showed the threshold used by ORFLine in the according metrics.

**Figure S4.** The canonical ORFs of dORF-containing transcripts may be translationally enhanced. Cumulative distribution of translation efficiency in expressed dORF-containing transcripts versus transcripts lacking dORFs as control. Significance was computed using a two-sample Kolmogorov–Smirnov test, P = 0.437.

**Figure S5.** Selected putative micropeptides are conserved by amino acid sequence. The amino acid sequence of the following micropeptides from mouse, human, chimpanzee, gorilla, cow and dog are compared: A) Phf21a (uORF) B) BC031181 C) Zdhhc5 (uORF) D) Tbpl1 (uORF) E) 1190007I07Rik F) Opa1 (uORF) G) Slc39a9 (uORF). Conservation was displayed as followed: * fully conserved : strongly similar . weakly similar.
